# Paroxysmal Symptoms As the First Manifestation of Multiple Sclerosis Mimicking a Transient Ischemic Attack: A Report of Two Cases

**DOI:** 10.3389/fneur.2017.00585

**Published:** 2017-11-03

**Authors:** Yao Zhang, Siyuan Fan, Fei Han, Yan Xu

**Affiliations:** ^1^Multiple Sclerosis Center, Department of Neurology, Peking Union Medical College Hospital, Peking Union Medical College, Chinese Academy of Medical Sciences, Beijing, China; ^2^Department of Neurology, Peking Union Medical College Hospital, Peking Union Medical College, Chinese Academy of Medical Sciences, Beijing, China

**Keywords:** paroxysmal symptoms, multiple sclerosis, transient ischemic attack, transcranial Doppler, mimic

## Abstract

Paroxysmal symptoms are unusual manifestations of multiple sclerosis (MS). When presented as the first clinical manifestation, paroxysmal symptoms may easily be mistaken for transient ischemic attack (TIA). Previously, several cases of MS that reported with paroxysmal symptoms were misdiagnosed as TIA. Here, we report two additional cases, focusing on the clinical characteristics of paroxysmal symptoms in MS. Both cases had paroxysmal symptoms as their first manifestation; one presented with transient dizziness, left face numbness, and right limb weakness, and the other presented with episodic lightheadedness, blurred vision, nausea, palpitations, and tremulousness upon standing. Both of the patients’ symptoms were mistaken for TIA at first, based on microembolic signals recorded by transcranial Doppler, but were later correctly diagnosed with MS based on neuroimaging and lumbar puncture. The paroxysmal symptoms responded to carbamazepine and were relieved completely after administration of intravenous methylprednisolone and interferon. Herein, we aim to summarize the differences between paroxysmal symptoms seen in MS and TIA, to facilitate a timely differential diagnosis and recommend an early appropriate treatment.

## Background

Multiple sclerosis (MS) is the most common immune-mediated inflammatory demyelinating disease of the central nervous system. The characteristic neuropathological feature of MS is the presence of focal demyelinated plaques within white matter of the central nervous system. Based on this pathological feature, the classic clinical manifestations of MS include optic neuritis, long tract symptoms (e.g., numbness or weakness), rather than transient symptoms as in epilepsy and transient ischemic attack (TIA). However, paroxysmal symptoms have occasionally been reported in MS since in 1959 (1). These transient symptoms are characterized by their brevity, frequency, stereotyped fashion, and response to carbamazepine (2). If untreated they tend to continue in clusters for days up to a few months. Paroxysmal symptoms in MS, especially when presented as the first clinical manifestation, could be mistaken for TIA, epilepsy, and neuralgias (3). Thus, identifying paroxysmal symptoms as a possible first clinical symptom of MS is important for differential diagnosis from TIA, as well as for early diagnosis and treatment of the disease. We report two cases of MS with paroxysmal symptoms as their first manifestation and review the literature. Both patients were misdiagnosed with TIA before receiving the proper diagnosis of MS.

## Case Presentation 1

A 32-year-old male developed episodic, transient, sudden-onset dizziness, left face numbness, and right limb weakness. He experienced the attacks 20–30 times a day, with each episode lasting 10–20 s. He was completely well between the attacks. The patient was admitted to our emergency department with the suspicion of TIA. Cardiovascular risk factors included cigarette smoking of 5–6 cigarettes/day for 10 years. There was no history of migraines, and his neurological exam was normal at admission.

## Description of Laboratory Investigations and Diagnostic Tests

**Figure 1:**
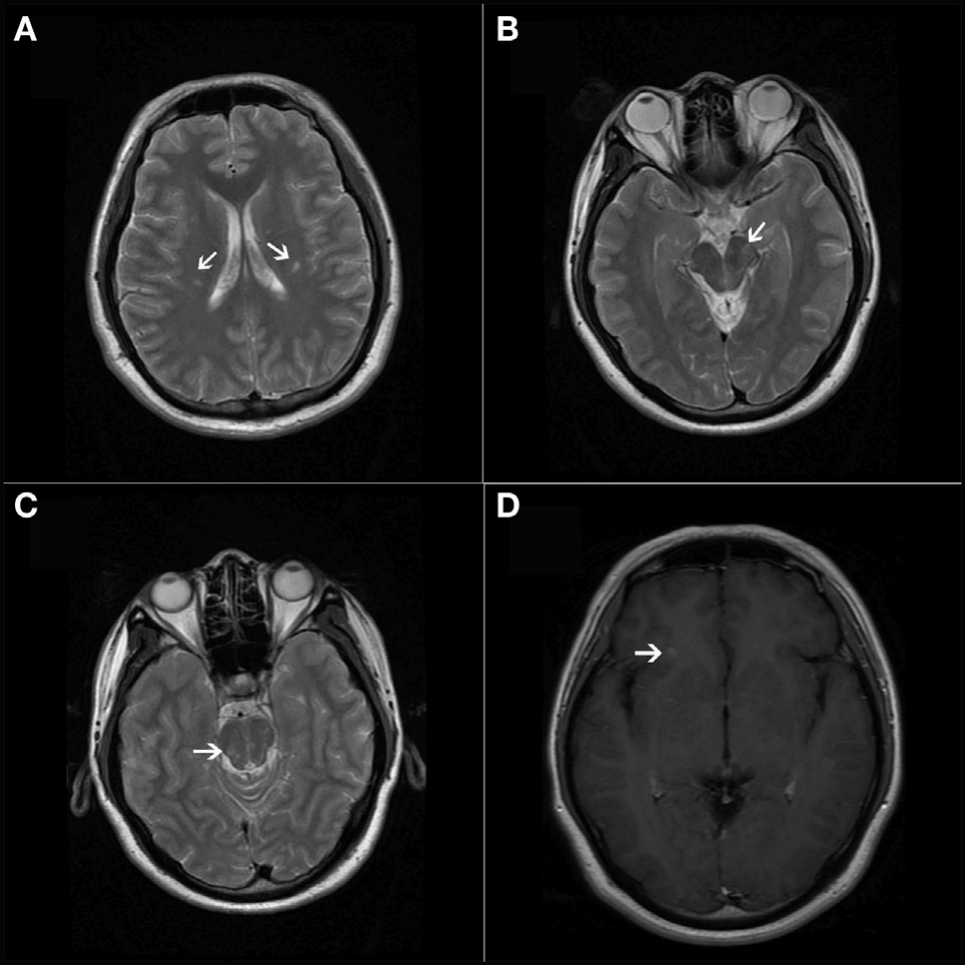
**(A–C)** T2-weighted axial magnetic resonance imaging showed lesions in bilateral periventricular and infratentorial areas of the CNS. **(D)** T1-weighted contrast-enhanced image showed a simultaneous presence of asymptomatic gadolinium-enhancing lesion at the juxtacortical area of the anterior part of the insular cortex.

A head CT excluded intracranial hemorrhage and subarachnoid hemorrhage. With the suspicion of TIA, extracranial, intracranial arteries, and the heart were studied. The cardiovascular examinations, including extra- and intracranial ultrasound and transthoracic echocardiography, were normal. Transcranial Doppler (TCD) contrast agent was used to detect intracardiac shunts, and three microembolic signals were recorded on TCD spectrum by 2 Hz probes within 25 s of contrast injection. The preliminary diagnosis was cryptogenic stroke with patent foramen ovale (PFO), and the patient was treated with aspirin (100 mg/day) for 7 days, but his episodes persisted. To exclude ischemic stroke and other related disease, brain magnetic resonance imaging (MRI), including six sequences (T1-weighted axial, T2-weighted sagittal, T2-weighted axial, FLAIR, DWI, and ADC) as well as T1-weighted contrast-enhanced images was done 1 week later. The MRI showed an asymptomatic gadolinium-enhancing lesion in the juxtacortical region of the frontal lobe on T1-weighted images with gadolinium enhancement. Several hyperintense lesions in the midbrain, pons, and periventricular region had also been found on T2-weighted images without gadolinium enhancement (Figure 1). The serum autoantibody test was negative, and connective tissue diseases were ruled out. During this episodes, his electroencephalogram was normal, and epileptic seizure was ruled out. The patient was suspected of demyelinating diseases, and lumbar punctures was done. Cerebrospinal fluid (CSF) cell counts and protein levels were normal, but isoelectric focusing revealed oligoclonal IgG bands (OCBs). All of these results were consistent with the McDonald criteria, and the attacks were qualified as paroxysmal symptoms of MS. The patient was treated with intravenous methylprednisolone (1,000 mg) for 5 days as well as carbamazepine (200 mg/day). His attacks promptly decreased to less than 10 times per day. He was subsequently started on interferon, and his attacks resolved completely 3 weeks later. The carbamazepine was discontinued after 3 months, and he was relapse-free during his 1-year follow-up. Unfortunately, he did not do follow-up MRI in our hospital after interferon was administered.

## Case Presentation 2

A 16-year-old female with no cardiovascular risk factors presented with episodic and sudden-onset lightheadedness, blurred vision, nausea, palpitations, and tremulousness upon standing. Syncope occurred several times when she stood up quickly. She experienced such episodes 7–10 times daily, each lasting less than 1 min. Her past medical history was clear, and her neurological exam was normal at admission.

## Description of Laboratory Investigations and Diagnostic Tests

**Figure 2:**
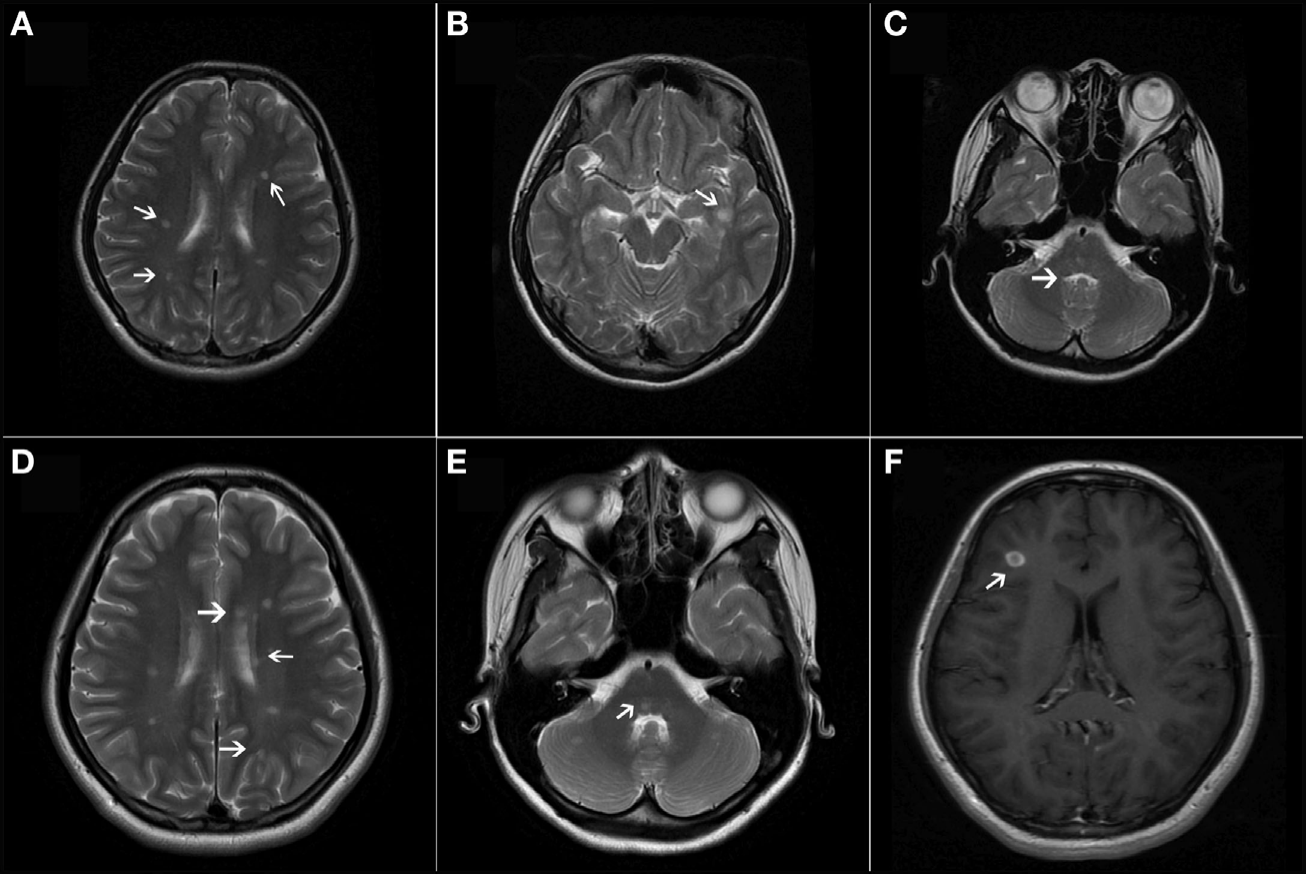
**(A–C)** T2-weighted axial magnetic resonance imaging (MRI) revealed multiple, ovoid-shaped, hyperintense lesions in the bilateral periventricular regions, the juxtacortical area of the left inferior temporal lobe, and the pons. **(D,E)** T2-weighted axial MRI showed new lesions in the periventricular regions and the cerebellum. **(F)** T1-weighted contrast-enhanced image showed one asymptomatic gadolinium-enhancing lesion in the right frontal lobe.

Since she was suspected of TIA at admission, MRI, carotid ultrasound, and TCD were done the next day. The T2-weighted MRI of her brain revealed multiple, ovoid-shaped, hyperintense lesions in the periventricular region, the inferior temporal lobe white matter, and the pons. No gadolinium-enhancing lesion was found on T1-weighted contrast-enhanced image. The carotid ultrasound was normal but 17 microembolic signals were recorded by 4 Hz probes on TCD spectrum within 6 s, while other cardiovascular examinations were normal. She was suspected of TIA, PFO, and was treated with aspirin (100 mg/day). However, she later experienced even more episodes, approximately 20–30 times per day. The episodes resolved gradually 2 months later, but after 6 months had passed, the episodes returned. A follow-up MRI revealed new T2-hyperintense lesions in characteristic locations (Figure 2): periventricular, juxtacortical, and infratentorial regions, as well as one gadolinium-enhancing lesion on the frontal lobe. MS was suspected, and lumbar puncture was done. The CSF total leukocyte count and protein level were normal but isoelectric focusing revealed OCBs different from bands in serum. In a head-up tilt table test, she demonstrated a sustained heart rate increase of 52 beats per minute (bpm) within the first 10 min of tilt (from 68 to 120 bpm) without orthostatic hypotension. She was diagnosed with MS and postural tachycardia syndrome. The lesion on the pons was responsible for her vegetative instability. Intravenous methylprednisolone (1,000 mg) was administered for 5 days, and carbamazepine (200 mg/day) was also started. Her paroxysmal symptoms resolved promptly, and 3 weeks later carbamazepine was stopped. She was subsequently started on interferon beta-1b (250 μg every other day). She reported that the same episodes recurred when she discontinued interferon half a year later. Since there were no other neurological symptoms or increased disability, steroid treatment was not initiated. Her episodes resolved again after continuation of interferon.

## Discussion

Paroxysmal symptoms are unusual manifestations of MS, especially at onset of the disease. An electronic case database was established at the MS clinic of the Department of Neurology, Peking Union Medical College Hospital in 2011 (4). By December 2016, a total of 520 MS patients had been included, yet only 4 (0.77%) cases presented with paroxysmal syndromes as their first clinical manifestation. This rate is comparable to the previous largest study that reported 1.1% (15/1,396) of MS patients presented with paroxysmal symptoms (5). A population-based, MS cohort study reported the yearly incidence of brainstem and spinal paroxysmal symptoms was 190 cases in 10,000 MS patients (6).

When paroxysmal symptoms are the presenting manifestation of MS, it can be challenging for clinicians to differentiate these symptoms from TIA. Several cases of paroxysmal symptoms in MS have been reportedly mistaken for TIA (7, 8). We found in agreement with these reports that paroxysmal symptoms in MS had several typical characteristics that can be differentiated from TIA (Table 1). Paroxysmal symptoms in MS are characterized by their sudden onset, brevity (usually seconds to minutes) (5), frequency (from 10 to 20 times per day up to a few hundred times per day), stereotyped fashion, and relatively long clinical course. If untreated, they tend to continue in clusters for days up to a few months before remission (7), coincident with a new episode of inflammatory demyelination. Relapses in MS have been defined as episodes of neurological disturbances last for at least 24 h. As a consequence, paroxysmal symptoms are accepted as relapses as long as they consist of multiple episodes occurring over not less than 24 h. These transient symptoms usually have a positive response to carbamazepine, while TIA does not.

**Table 1:** Comparison of clinical manifestations between paroxysmal symptoms and transient ischemic attack (TIA).

	Paroxysmal symptoms	TIA
Trigger	Can be triggered by sensory stimuli, hyperventilation, or movement (7)	Decreased perfusion pressure

Brevity	Lasting seconds to minutes	Lasting minutes to hours

Frequency	From 10 to 20 times per day up to a few hundred times per day	Less than 10 times per day, even once per week

Clinical course	Tend to continue in clusters with great intensity for days up to a few months. Followed by remission	Continue for days and sometimes proceed to stroke

Response to carbamazepine	Good response	No response

Patent foramen ovale, which can be detected in about 25% of the healthy population, is a common anatomical variant leading to right-to-left shunt (RLS) (9). Compared to transesophageal echocardiography (TEE), the gold standard of RLS detection, TCD is a faster, less expensive, non-invasive bedside method for the detection of RLS in patients with cryptogenic cerebral ischemia and TIA. The sensitivity and specificity of TCD in the detection of PFO have been reported to be 96.1 and 92.4%, respectively. Compared to TCD, TEE is superior in terms of higher positive likelihood ratio values, which suggests that TCD is less specific for the detection of PFO (10). TCD diagnoses right-to-left atrial shunt (RLS) by detection of microbubbles (MB) passing through the middle cerebral artery. The threshold value of 42 MB during normal breathing was suggested to discriminate shunts related to stroke or TIA from a “bystander PFO” with no pathophysiological relationship to the symptoms (11). Both of the two cases in this report had considerably less than 42 MB (3 MB in case 1, and 17 MB in case 2) detected by TCD, and the RLS had no clinical significance.

Several clinical types of paroxysmal symptoms have been described (3), including paroxysmal dysarthria with ataxia, paroxysmal diplopia, paroxysmal paresthesia, tonic spasm, and paroxysmal hemiparesis. Different clinical manifestations reflect the disruption of axonal conduction in different areas of the central nervous system. The mechanisms of paroxysmal symptoms in MS are not fully understood, but are likely mediated by ephaptic transmission (12). On this basis, membrane-stabilizing drugs are effective in almost all patients (7). The paroxysmal symptoms have a tendency to occur in the early stages of the MS disease course (2). A possible explanation for this is that only the intact naked axons that exist in the early stages of MS and that feature relatively less inflammatory damage could create paroxysmal symptoms, by spreading the neuronal conduction transversely and activating adjacent anatomical structures (2).

## Conclusion

Paroxysmal symptoms that present as the first manifestation of MS may easily be diagnosed as TIA. Paroxysmal symptoms in MS can be differentiated from TIA by their brevity (usually seconds to minutes), frequency (10–20 times per day up to a few hundred times per day), relatively long-clinical course (tend to continue in clusters for days up to a few months), and positive response to carbamazepine. Differential diagnosis of MS cases presented with paroxysmal symptoms from TIA is important for early diagnosis of MS and early initiation of the disease-modifying therapies.

## Ethics Statement

No interventions were performed outside routine clinical care for these two patients. No formal research ethics approval was required because this is a case report and there was no experimental intervention into routine care. This study was carried out in accordance with the recommendations of Ethics Committee of Peking Union Medical College Hospital with written informed consent from all subjects. All subjects gave written informed consent to participate in the study in accordance with the Declaration of Helsinki. The protocol was approved by the Ethics Committee of Peking Union Medical College Hospital. Written informed consent was obtained from all the participants for the publication of this case report.

## Author Contributions

SF and FH conducted the patients’ investigations and provided clinical care, reviewed the manuscript, and approved the final manuscript as submitted. YZ planned the case report, drafted the initial manuscript, reviewed and revised the manuscript, and approved the final manuscript as submitted. YX helped plan clinical investigations, critically reviewed and revised the manuscript, and approved the final manuscript as submitted.

## Conflict of Interest Statement

The authors declare that the research was conducted in the absence of any commercial or financial relationships that could be construed as a potential conflict of interest.
